# Physiological Responses of *Kosteletzkya virginica* to Coastal Wetland Soil

**DOI:** 10.1155/2015/354581

**Published:** 2015-03-11

**Authors:** Hongyan Wang, Xiaoli Tang, Honglei Wang, Hongbo Shao

**Affiliations:** ^1^Key Laboratory of Coastal Biology & Bioresources Utilization, Yantai Institute of Coastal Zone Research (YIC), Chinese Academy of Sciences (CAS), Yantai 264003, China; ^2^Yantai Academy of China Agriculture University, Yantai 264670, China; ^3^University of Chinese Academy of Sciences, Beijing 100049, China; ^4^Institute of Biotechnology, Jiangsu Academy of Agricultural Sciences, Nanjing 210014, China

## Abstract

Effects of salinity on growth and physiological indices of *Kosteletzkya virginica* seedlings were studied. Plant height, fresh weight (FW), dry weight (DW), and net photosynthetic rate (*P*_*n*_) increased at 100 mM NaCl and slightly declined at 200 mM, but higher salinity induced a significant reduction. Chlorophyll content, stomatal conductance (*G*_*s*_), intercellular CO_2_ concentration (*C*_*i*_), and transpiration rate (*E*) were not affected under moderate salinities, while markedly decreased at severe salinities except for the increased *C*_*i*_ at 400 mM NaCl. Furthermore, no significant differences of *F*_*v*_/*F*_*m*_ and ΦPSII were found at lower than 200 mM NaCl, whereas higher salinity caused the declines of *F*_*v*_/*F*_*m*_, ΦPSII, and qP similar to *P*_*n*_, accompanied with higher NPQ. Besides, salt stress reduced the leaf RWC, but caused the accumulation of proline to alleviate osmotic pressure. The increased activities of antioxidant enzymes maintained the normal levels of MDA and relative membrane permeability. To sum up, *Kosteletzkya virginica* seedlings have good salt tolerance and this may be partly attributed to its osmotic regulation and antioxidant capacity which help to maintain water balance and normal ROS level to ensure the efficient photosynthesis. These results provided important implications for *Kosteletzkya virginica* acting as a promising multiuse species for reclaiming coastal soil.

## 1. Introduction

Soil salinization adversely affects crop growth and productivity and it has become a global ecological and resource problem [1, 2]. It is estimated that more than 800 million hectares of land has been affected by salinity in the world, equating to 6% of the world's total land area [3]. The area of saline land is still expanding because of the changes in environment and irrational exploitation [4, 5] and this has become a serious threat to the sustainable development of agriculture because most crops have only a low degree of salt resistance [6]. In order to expand cultivated land and increase food production, it is very necessary to ameliorate and reclaim the great area of salt-affected land, especially barren coastal land resources. In recent years, growing salt-tolerant plants with economic values has proven to be an effective method for alternative agricultural production and for revegetation in saline farmland or salt-affected coastal zone [7–10]. As known, there exist some halophytes which can thrive in high-salt environments such as coasts, wetlands, and inland deserts. These species have evolved complicated mechanisms at different levels (molecular, cellular, and whole plant) that enable them to successfully cope with these adverse conditions. They can be either domesticated into new, salt-tolerant crops or used as a potent genetic source for the improvement of salt tolerance in conventional crops through genetic engineering [11–13]. However, different plant species and genotypes within species have different growth and physiological responses to the given salinity range and duration. So it is very important to screen salt-tolerant plants with economic values for saline agriculture as well as to know about their degree of salt tolerance and mechanisms of resistance to provide useful information for sustainable saline soil amelioration and saline agriculture [14, 15].

*Kosteletzkya virginica* (L.), also commonly known as seashore mallow, is a perennial facultative halophytic species in Malvaceae family, natively distributing in coastal areas from Long Island along the Atlantic coast of the US west to eastern Texas, and is also found in coastal areas of Eurasia [16, 17]. Seashore mallow grows frequently in seashore soil containing 0.3 to 2.5% sodium salt (mainly NaCl) [18] and also survives in waterlogged soil [19]. Because of its economic values and the tolerance to saline soil, this species has been introduced in China and recommended as a potential cash crop for alternative saline agriculture [8, 20, 21]. During the past two decades, researches on seashore mallow mostly focused on ecological adaptation, safety from invasiveness, and economic benefits and proved it to be a promising species for numerous purposes, such as food, fodder, biofuel, fiber, health care, and ornamental [12]. Although seashore mallow has been shown to be salt tolerant, its range of salt tolerance and underlying physiological mechanisms of tolerance are largely unknown. So this work is aimed at studying salt-induced changes in the growth and physiological attributes of* Kosteletzkya virginica* (L.) which can not only disclose its range of salt tolerance but also help us understand its physiological mechanisms of salt tolerance.

## 2. Materials and Methods

### 2.1. Plant Material, Growth Condition, and Stress Treatments

The seeds of* Kosteletzkya virginica* were collected from the Yellow River delta coastal wetland ecological experimental station located in Dongying, Shandong, China. The seeds were soaked in concentrated sulfuric acid for 20 min to remove the hard shell over and then thoroughly rinsed with deionized water. The seeds were then sown in plastic pots (18 cm diameter × 13 cm depth) containing washed sand and all the pots were daily watered with 1/2 Hoagland nutrient solution and well drained with holes at the bottom. All pots were placed outdoors and were kept out of rain. Temperatures during the experiment were 25–29°C during the day and 18–22°C at night. Five weeks after emergence, five uniform seedlings with eight leaves (about 30 cm height) were kept in each pot and were subjected to salt stress treatments. Within the salt-stress group, NaCl was added to 1/2 Hoagland nutrient solution to provide four levels of salinity: 100, 200, 300, and 400 mM/L. Every 3 pots (5 seedlings/pot) were used by different treatments; therefore 15 seedlings per treatment were considered as multiple replicates. Control plants were maintained by watering with 1/2 Hoagland nutrient solution and the salt-stress plants were watered with 1/2 Hoagland nutrient solution containing the appropriate NaCl once daily around 17:00–18:00 h. The high NaCl concentration (>100 mM) was imposed incrementally by 100 mM step every day until final concentrations were reached. Seedlings of each treatment were treated with 1/2 Hoagland nutrient solution of final NaCl concentration for 14 days. The third fully expanded leaves from the top were used for measuring photosynthetic and fluorescent parameters. Leaves and roots in each treatment were harvested, weighed, frozen in liquid nitrogen, and stored at −80°C in a freezer for the subsequent experiments.

### 2.2. Growth Parameters

At the end of the experiment, the plant height of seedlings (from the sand surface to the tip of the main stem) was measured. All materials were rinsed three times in distilled water, dried with filter paper, and weighed (fresh weight, FW). For dry weight (DW), plants were dried at 80°C for 48 h.

### 2.3. Chlorophyll Content

Leaf samples (0.2 g fresh weight) were soaked in 20 mL 95% (v/v) ethanol at 4°C in darkness until the tissues became totally white. Extracts were used to measure the absorbance at 649 nm and 665 nm; the chlorophyll content was calculated according to Hartmut [22].

### 2.4. Gas Exchange and Chlorophyll Fluorescence

Gas exchange and chlorophyll fluorescence were simultaneously detected using an open photosynthetic system (LI-6400XT, Li-Cor, USA) equipped with a fluorescence leaf chamber (6400-40 LCF, Li-Cor, USA). The leaves were dark-adapted for 30 min before the measurements. The minimal fluorescence level in the dark-adapted state (*F*_*o*_) was measured using a modulated pulse (<0.05 *μ*mol photons m^−2^ s^−1^ for 1.8 s). Maximal fluorescence (*F*_*m*_) was measured after applying a saturating actinic light pulse of 8000 *μ*mol photons m^−2^ s^−1^ for 0.7 s. Subsequently, actinic light intensity was altered to 1000 *μ*mol photons m^−2^ s^−1^ in leaf cuvette and then maintained for about 30 min. The temperature, carbon dioxide (CO_2_) concentration, and relative humidity in the leaf cuvette depended on ambient conditions. Stomatal conductance (*G*_*s*_) and intercellular CO_2_ concentration (*C*_*i*_) were recorded simultaneously with *P*_*n*_. In addition, steady-state fluorescence yield (*F*_*s*_) was also recorded. A saturating actinic light pulse of 8000 *μ*mol photons m^−2^ s^−1^ for 0.7 s was then used to produce maximum fluorescence yield (*F*_*m*_′) by temporarily inhibiting photosystem II (PSII) photochemistry. Using fluorescence parameters determined in both light- and dark-adapted states, the actual photochemical efficiency of PSII (ΦPSII) proportion of open PSII (qP) and nonphotochemical quenching (NPQ) was calculated [23].

### 2.5. Relative Water Content (RWC)

The RWC was measured according to Flexas et al. [24]. Fresh leaves were harvested and weighed (called fresh weight, FW), then soaked in deionized water for 24 h at 4°C, and weighed (called saturated fresh weight, SFW). Finally, the leaves were dried completely in an oven and weighed (called dry weight, DW). RWC was calculated as RWC = (FW − DW)/(SFW − DW).

### 2.6. Proline Content

Leaf samples (0.2 g fresh weight) were homogenized using pestle and mortar with 3 mL of 5% (w/v) sulphosalicylic acid and incubated at 100°C for 10 min. After centrifugation at 13000 g for 10 min, 2 mL glacial acetic acid and 3 mL ninhydrin reagent were added to 2 mL of the supernatant and incubated at 100°C for 40 min. After cooling to room temperature, 5 mL toluene was added to the mixture and the absorbance at 520 nm of the toluene phase was recorded. Proline concentration was determined from a standard curve and calculated on fresh weight basis. The standard curve was plotted according to the proline solution of known concentration.

### 2.7. Relative Membrane Permeability

Relative membrane permeability was estimated by the extent of electrolyte leakage (EL) measured by a conductivity meter (DDS-11A, China) according to Yang et al. [25]. Leaf samples (0.2 g fresh weight and cut into pieces) were submerged into 20 mL distilled water and kept at room temperature for 2 h. Then, the initial electrical conductivity of each sample (EC_1_) was measured. The samples were then boiling at 100°C for 10 min and cooled to room temperature and the final electrical conductivity (EC_2_) was measured. The electrical conductivity of distilled water was marked as EC_0_. The relative membrane permeability was calculated as the following formula:

(1) Relative  membrane  permeability%=EC1−EC0EC2−EC0×100.

### 2.8. Determination of Lipid Peroxidation

The level of lipid peroxidation was indicated by malondialdehyde (MDA) content. Leaf samples (0.2 g fresh weight) were ground under liquid nitrogen and then homogenized in 3.5 mL 200 mM phosphate buffer solution (pH 7.8). After centrifugation at 4°C and 12000 g for 10 min, 1 mL of the supernatant was added to 0.5% thiobarbituric acid (TBA) in 20% trichloroacetic acid (TCA). The mixture was incubated in water bath at 95°C for 30 min and then cooled in an ice bath to stop the reaction. After centrifugation at 10000 g for 10 min, the absorbance of supernatant was measured at 532 nm, 600 nm, and 450 nm. The concentration of MDA was calculated as MDA content (*μ*M) = 6.45(A532–A600) − 0.56A450.

### 2.9. Antioxidant Enzyme Extraction and Activity Assay

Leaf samples (0.2 g) were ground with liquid nitrogen and then homogenized in 5 mL of 50 mM phosphate buffer (pH 7.8) containing 1 mM EDTA and 2% (w/v) PVP. The homogenate was centrifuged at 4°C and 13000 g for 10 min and the supernatant was used in the following enzyme activity assays. Superoxide dismutase (SOD) activity was assayed by measuring the inhibition in photoreduction of nitroblue tetrazolium (NBT) following the method described by Beyer Jr. and Fridovich [26]. One unit of SOD activity was defined as the amount of enzyme required to cause a 50% inhibition of the NBT photoreduction rate at 560 nm. The reaction mixture contained 0.3 mL riboflavin (13 *μ*M), 0.3 mL L-methionine (130 mM), 0.3 mL NBT (63 *μ*M), and 2.1 mL enzyme extract. Catalase (CAT) activity was determined directly as a decrease in the absorbance at 240 nm for 1 min following the decomposition of H_2_O_2_ in a reaction mixture composed of 2 mL 15 mM H_2_O_2_ and 1 mL enzyme extract [27]. Peroxidase (POD) activity was evaluated from the rate of guaiacol oxidation at 470 nm [28]. The reaction mixture included 0.1 mL of enzyme extract and 2.9 mL phosphate buffer (100 mM, pH 7.0) containing 20 mM guaiacol and 2% H_2_O_2_. The absorbance at 470 nm was measured at 1 min intervals for 5 min. An increase in the absorbance (0.01 unit/min) was equated to one unit of POD activity. The activities of SOD, CAT, and POD were expressed as units per milligram of fresh weight.

### 2.10. Statistical Analysis

One-way ANOVA was performed using SPSS computer package (SPSS Inc., USA) for all sets of data. Significant differences between means were determined through LSD test. Differences were considered statistically significant when *P* < 0.05. All data were presented as mean ± SD.

## 3. Results

### 3.1. Effects of Salt Stress on Growth and Chlorophyll Content

**Figure 1:**
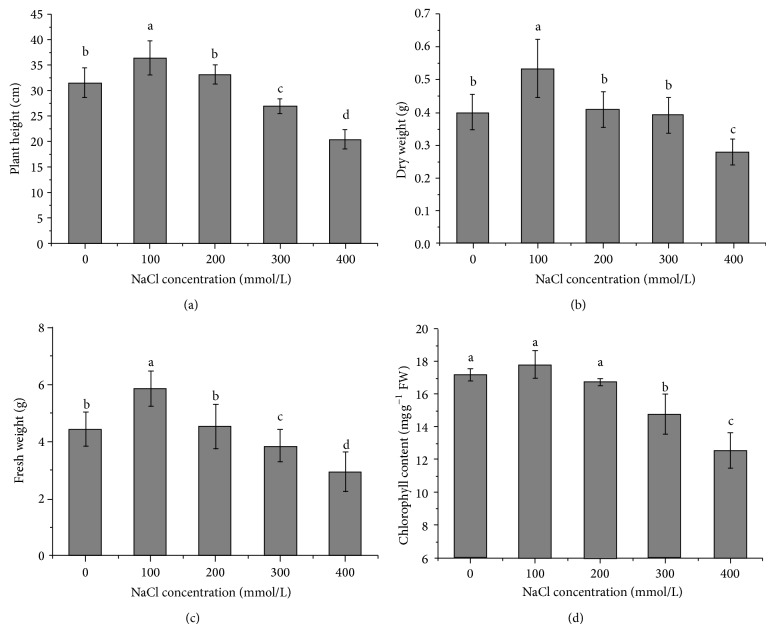
Changes of plant height (a), dry weight (b), fresh weight (c), and chlorophyll content (d) in seedlings exposed to different NaCl concentrations.

The responses of plant height, dry weight, and fresh weight had the similar trends (Figures 1(a), 1(b), and 1(c)). Mild salinity (100 mM NaCl) caused a significant increase in these indices, while moderate salinity (200 mM NaCl) had no significant influence. When exposed to high salinity (300 mM or more), all these indices were significantly decreased and accompanied with shorter internode length and smaller leaf area. In particular, at 400 mM NaCl treatment for 14 days, the values of them were reduced by 35.39%, 30.59%, and 33.61%, respectively, and most of the seedlings became yellow and withered. The chlorophyll content was unaffected before the salinity reached 200 mM NaCl but declined markedly at higher salinity than 300 mM NaCl.

### 3.2. Effects of Salt Stress on Gas Exchange and Chlorophyll Fluorescence

**Figure 2:**
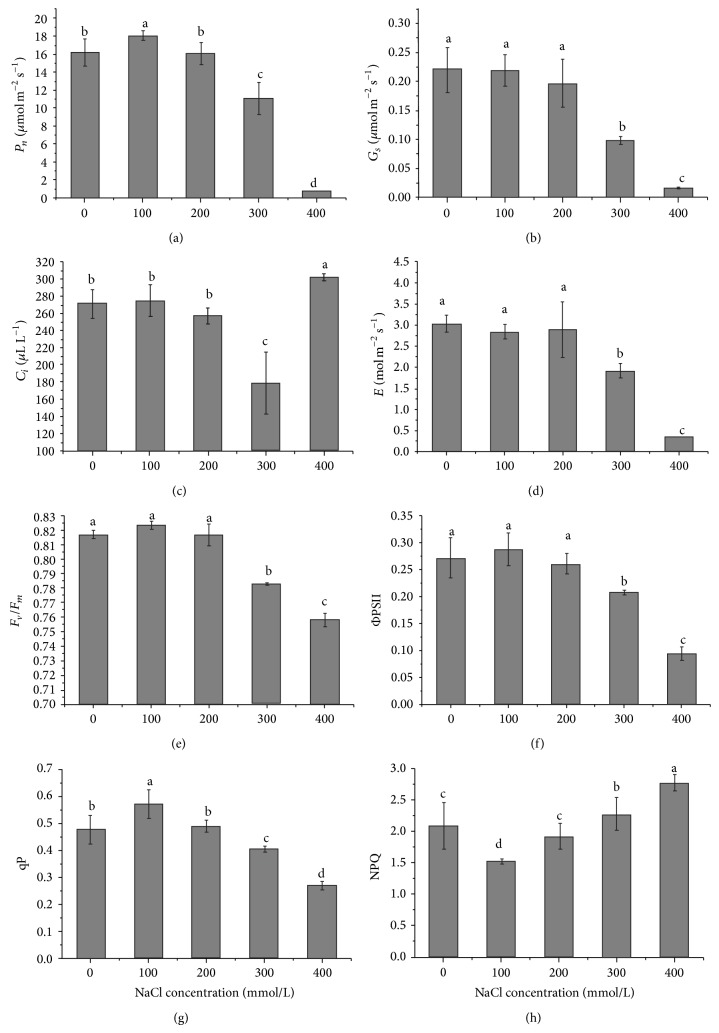
Changes of net photosynthetic rate (*P*_*n*_, (a)), stomatal conductance (*G*_*s*_, (b)), intercellular CO_2_ concentration (*C*_*i*_, (c)), transpiration rate (*E*, (d)), maximal photochemical efficiency of PSII (*F*_*v*_/*F*_*m*_, (e)), actual photochemical efficiency of PSII (ΦPSII, (f)), proportion of open PSII (qP, (g)), and nonphotochemical quenching (NPQ, (h)) in seedlings exposed to different NaCl concentrations.

The change in trend of *P*_*n*_ was similar to the changes of the growth parameters. *P*_*n*_ was promoted by low salinity (100 mM NaCl) and unaffected by moderate salinity (200 mM NaCl) but sharply declined at high salinity (300 mM or more). At 400 mM NaCl, photosynthesis was severely inhibited and *P*_*n*_ was almost reduced to zero. In addition, *G*_*s*_, *C*_*i*_, and *E* under moderate salinity (200 mM or less) were not affected and kept the same level as compared with those of the controls. When salinity was greater than 300 mM NaCl, *G*_*s*_ and *E* markedly decreased, but *C*_*i*_ firstly declined at 300 mM NaCl and then sharply increased at 400 mM NaCl. Furthermore, the responses of *F*_*v*_/*F*_*m*_ and ΦPSII showed the similar trend. No significant differences of *F*_*v*_/*F*_*m*_ and ΦPSII were found at lower than 200 mM NaCl, while higher salinity caused the declines of *F*_*v*_/*F*_*m*_, ΦPSII, and qP similar to *P*_*n*_, accompanied with higher NPQ (Figure 2).

### 3.3. Effects of Salt Stress on Relative Water Content (RWC) and Proline Content

**Figure 3:**
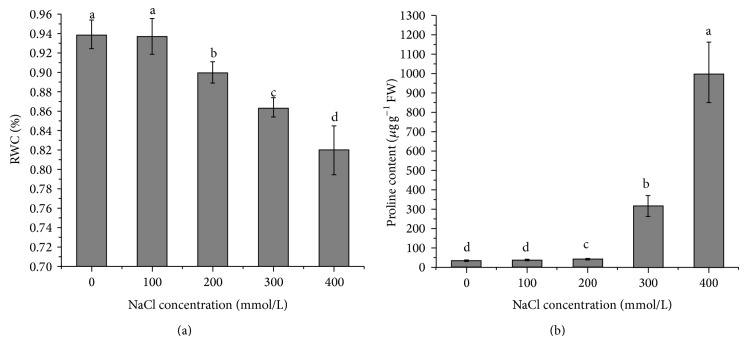
Changes of relative water content (RWC, (a)) and proline content (b) in seedlings exposed to different NaCl concentrations.

The leaf RWC was significantly reduced by salinities (higher than 100 mM NaCl) (Figure 3(a)). On the contrary, proline in leaves markedly accumulated (Figure 3(b)); when NaCl concentration was more than 200 mM especially, the leaf proline contents at 300 mM and 400 mM NaCl were approximately 9 times and 27 times higher than in the control, respectively.

### 3.4. Effects of Salt Stress on Lipid Peroxidation, Relative Membrane Permeability, and Antioxidant Enzyme Activities

**Figure 4:**
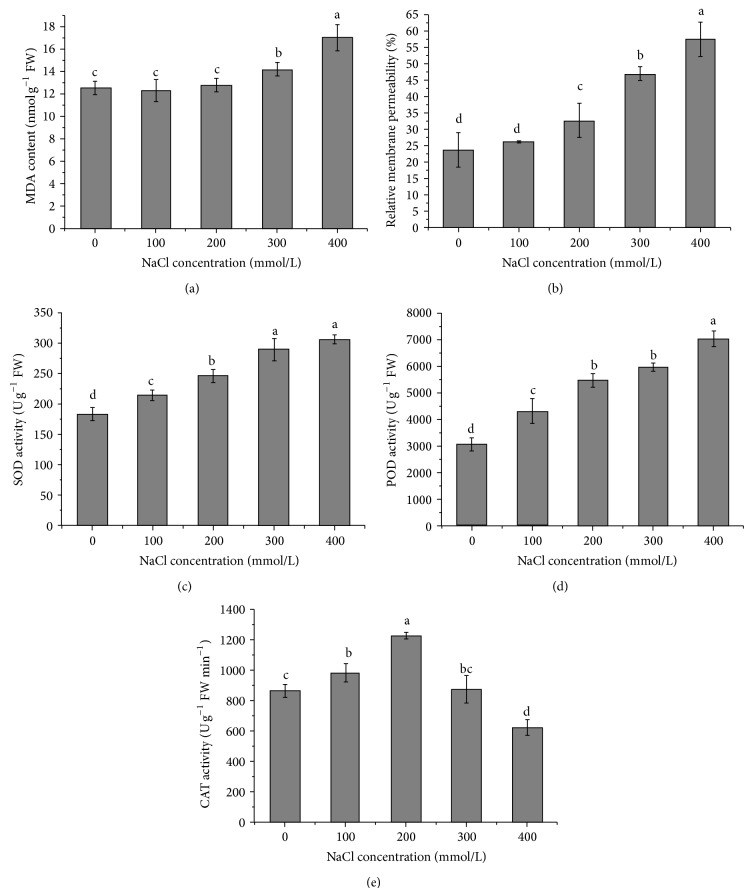
Changes of MDA (a), relative membrane permeability (b), SOD activity (c), POD activity (d), and CAT activity (e) in seedlings exposed to different NaCl concentrations.

Salt stress induced a significant increase in the levels of MDA (Figure 4(a)), indicating the generation of lipid peroxidation. A slight increase in the MDA content was observed under 200 mM NaCl, but 300 and 400 mM NaCl treatments caused a 13.09% and 35.91% increase in MDA content, respectively. These results were concomitant with a significant increase in relative membrane permeability (Figure 4(b)). There was also a gradual increase in SOD and POD activity (Figures 4(c) and 4(d)) with the increasing NaCl concentrations, but the activity of CAT was firstly stimulated by 100 mM NaCl and reached the maximum at 200 mM NaCl and then declined.

## 4. Discussions

Biomass change is the reflection of the comprehensive effects of salt stress on plant and it is widely used to evaluate a plant's salt tolerance. Generally, the response to salinity in plant is the reduced growth and photosynthesis [28–31], but some halophytes can survive and complete their life cycle in high-salt environments. Moreover, some even require a certain amount of salt to exhibit their maximum growth potentials. The results of our experiment also seemed to confirm this point.

Exposed to different NaCl concentrations for 14 days, the plant height, fresh weight (FW), and dry weight (DW) of* Kosteletzkya virginica* seedlings were obviously stimulated at 100 mM NaCl and slightly declined at 200 mM NaCl but higher NaCl concentrations induced a significant reduction in these indices.

The change of biomass is closely related to the change of net photosynthetic rate (*P*_*n*_) and *P*_*n*_ usually decreases with rising stress intensity. However, we found that *P*_*n*_ was enhanced at 100 mM NaCl and almost unaffected at 200 mM NaCl but declined sharply under salinities greater than 300 mM NaCl, especially at 400 mM NaCl; the value was close to zero. These changes of *P*_*n*_ might be positively correlated with chlorophyll content and leaf area under different salinities (Figure 1(d)) which is responsible for photosynthesis on one hand and on the other hand might be ascribed to the changes of *G*_*s*_. Under moderate salinity lower than 200 mM NaCl, *G*_*s*_ was not affected to keep constants *C*_*i*_ and *E*, but *G*_*s*_ and *E* were markedly decreased by increasing salinities. The reduction of *G*_*s*_ may reduce water loss through decreased transpiration to resist osmotic stress caused by salinity but also inevitably reduce *C*_*i*_ and restricted CO_2_ availability to limit *P*_*n*_. However, the exception was the rapid increase of *C*_*i*_ in contrast with the decreased *G*_*s*_ at 400 mM NaCl. It seemed to indicate that nonstomatal limitations had prevailed over stomatal limitations for the reduced *P*_*n*_. This could be confirmed by further measurements of chlorophyll fluorescence which provides useful information about PSII efficiency under salinity [32, 33]. No significant differences of *F*_*v*_/*F*_*m*_ and ΦPSII were found at lower than 200 mM NaCl, suggesting that PSII efficiency was not obviously damaged, whereas higher salinity caused the declines of *F*_*v*_/*F*_*m*_, ΦPSII, and qP similar to *P*_*n*_, accompanied with higher NPQ. These results showed that PSII had been damaged at higher salinity and the excess of absorbed excitation energy was dissipated as heat instead of participating in photochemical reaction.

Osmotic stress caused by salinity leads to the reduction of leaf relative water content and water potential [34, 35]. Accordingly, plants not only reduce water loss by adjusting the stomatal conductance and transpiration rate, but also enhance water uptake by osmotic adjustment which can be mediated through accumulation of inorganic ions and compatible solutes. Proline is one of the most important compatible solutes in halophytes which not merely plays an osmoregulatory function but also helps in scavenging ROS and stabilizing membrane, protein, and enzyme. Some researches indicated that salinity caused an increase of proline content in most plants [36–39]. Our results also supported the abovementioned. The leaf RWC was almost unchanged at moderate salinity (lower than 200 mM NaCl) and this was consistent with constant *G*_*s*_ and *E* under the same salinity. Higher salinities led to the decreased RWC which subsequently caused a sharp reduction of *G*_*s*_ accompanied by decreased *E*. So the reduction of *G*_*s*_ is considered as an adaptive character to reduce water loss from the leaves under salt stress. Apart from the reduction of *G*_*s*_, the accumulation of proline was also observed in our study. In particular, at 400 mM NaCl, the level of proline was 27 times higher than that in control.

Oxidative stress is another detriment caused by salinity. Some research demonstrated that decreased photosynthesis under salt stress caused the overproduction of ROS in plants which leads to lipid peroxidation, protein degradation, electrolyte leakage, and ultimately cell death. In order to scavenge the increased ROS, plants enhance their antioxidant defense systems involving nonenzymatic and enzymatic antioxidants [40]. The activities of antioxidant enzymes are closely related to antioxidant defense and salt tolerance in plants. However, these enzyme activities vary in different plant species. Some reports showed that the activities of antioxidant enzymes increased in salt-tolerant species whereas salt-sensitive species failed to do so. In our study, no significant changes of MDA level and electrolyte leakage were found under moderate salinity (lower than 200 mM NaCl), suggesting its membrane stability and better adaption, but higher salinity (higher than 300 mM NaCl) led to the accumulation of MDA, representing the increase of lipid peroxidation and membrane permeability. Meanwhile, the enzyme activities of SOD and POD continued to rise in the given salinity range, but the activity of CAT was firstly stimulated by 100 mM Nail and reached the maximum at 200 mM NaCl and then declined at 400 mM NaCl. So we speculated that moderate salinity caused oxidative damage, but the enhanced enzyme activities effectively scavenged the increased ROS immediately and maintained cell membrane stability. Once the accumulation of ROS caused by severe salinity exceeded the scavenging abilities of the antioxidant enzymes, a series of oxidative damages happened, such as damage to cell membrane, chlorophyll degradation, inhibition of enzyme activity, and photoinhibition.

In summary, our study showed that* Kosteletzkya virginica* seedlings have good salt tolerance and maintain relatively high biomass under moderate salinity levels (200 mM NaCl or less). The mechanisms for its salt tolerance may be partly attributed to osmotic regulation and antioxidant capacity and this in turn contributes to maintaining water balance and normal ROS level which subsequently ensure the efficient photosynthesis. Because the mechanisms for salt tolerance are complicated and largely unknown, there is still a lot of work to be done in order to elucidate the integrated mechanisms for salt tolerance operating in* Kosteletzkya virginica* seedlings, for example, how to keep nutritional balance and alleviate ion toxicity under salinity.
